# Supplementary figures and images for: DNA methylation-mediated modulation of rapid desiccation tolerance acquisition and dehydration stress memory in the resurrection plant Boea hygrometrica
Source: PLoS Genet. 2021 Apr 30;17(4):e1009549. doi: 10.1371/journal.pgen.1009549 (PMC8115786; doi:10.1371/journal.pgen.1009549)

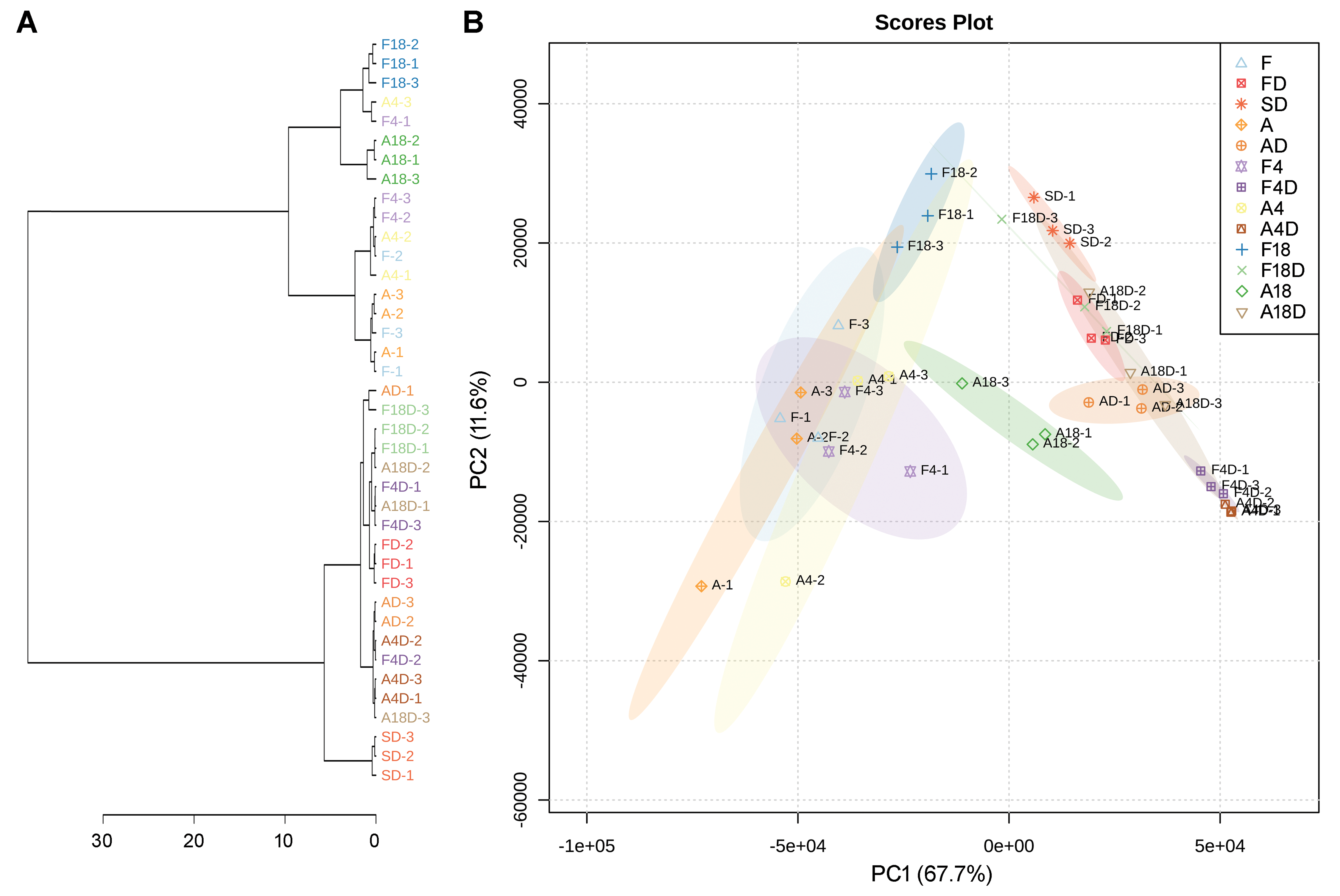

Supplement: S1 Fig — (A) HCA dendrogram showing the discrimination among samples based on the transcriptome data. (B) Score scatter plot of PCA showing the discrimination among samples based on the transcriptome data. The first principal component (PC1) and the second principal component (PC2) explained 67.7% and 11.6% of the variation in the initial data sets, respectively. (TIF) [file pgen.1009549.s001.tif]

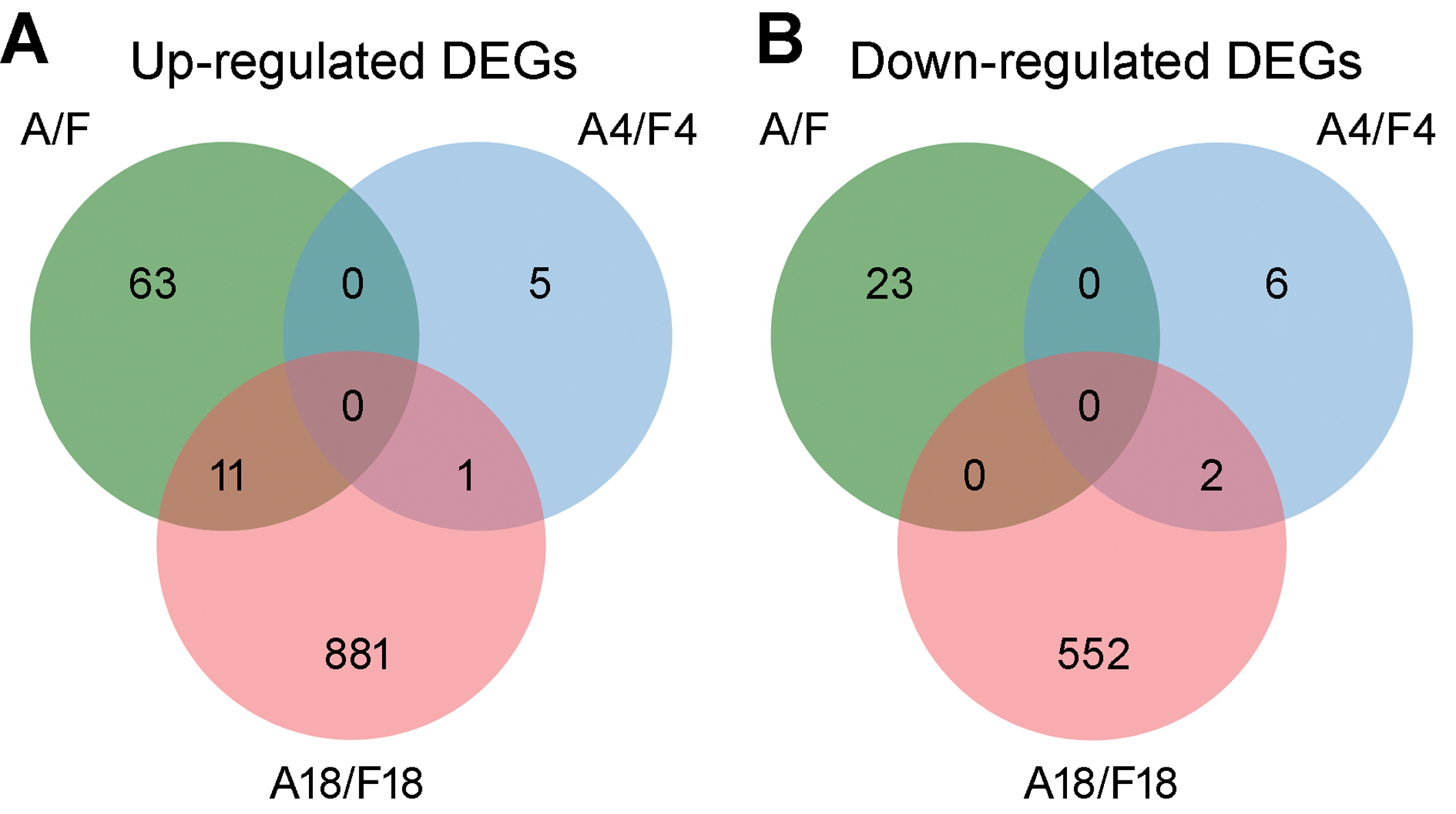

Supplement: S2 Fig — Venn diagrams displaying the overlap of up- (A) and down-regulated (B) DEGs between the acclimated and non-acclimated fresh plants at three growth stages. (TIF) [file pgen.1009549.s002.tif]

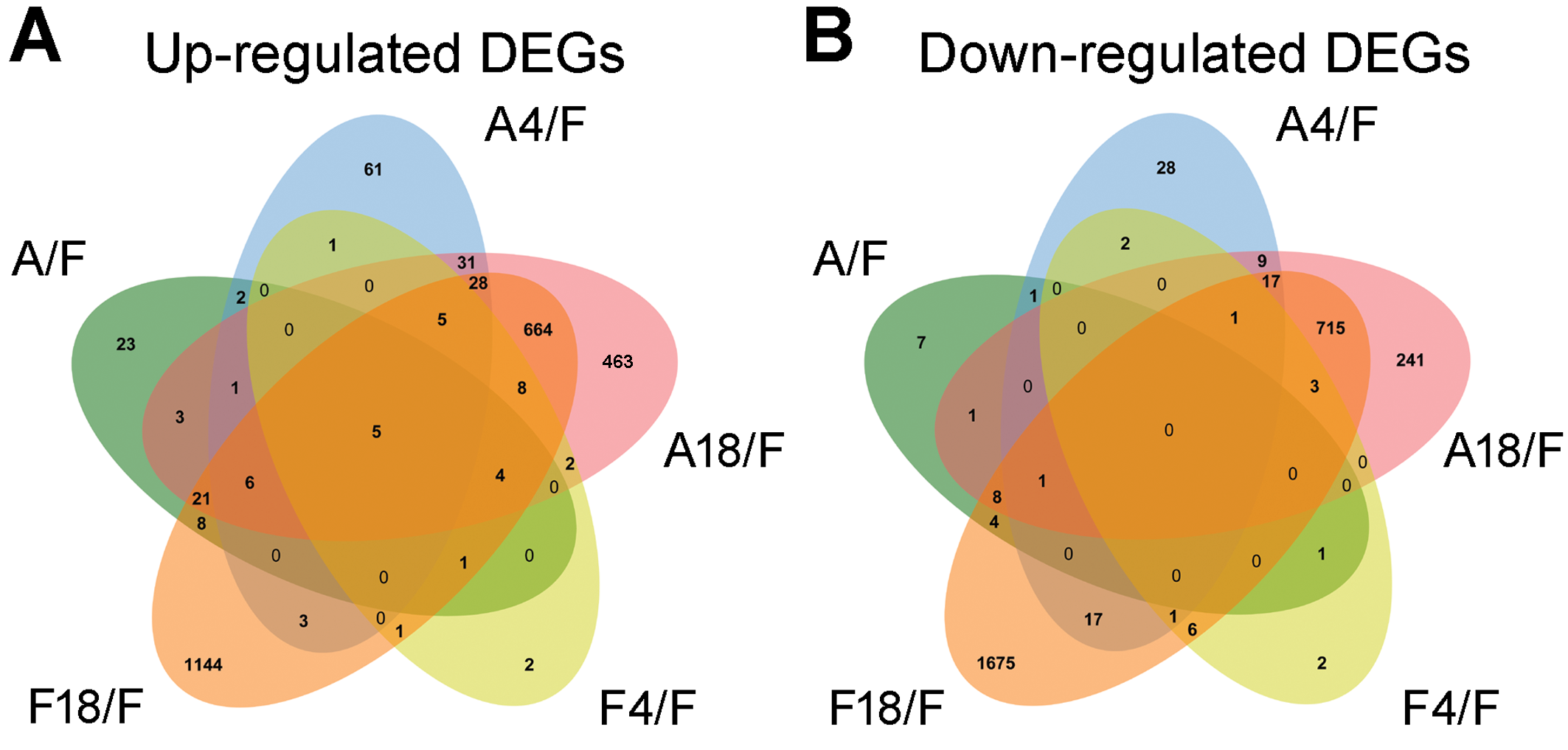

Supplement: S3 Fig — Venn diagrams displaying the overlap of up- (A) and down-regulated (B) DEGs during plant growth. (TIF) [file pgen.1009549.s003.tif]

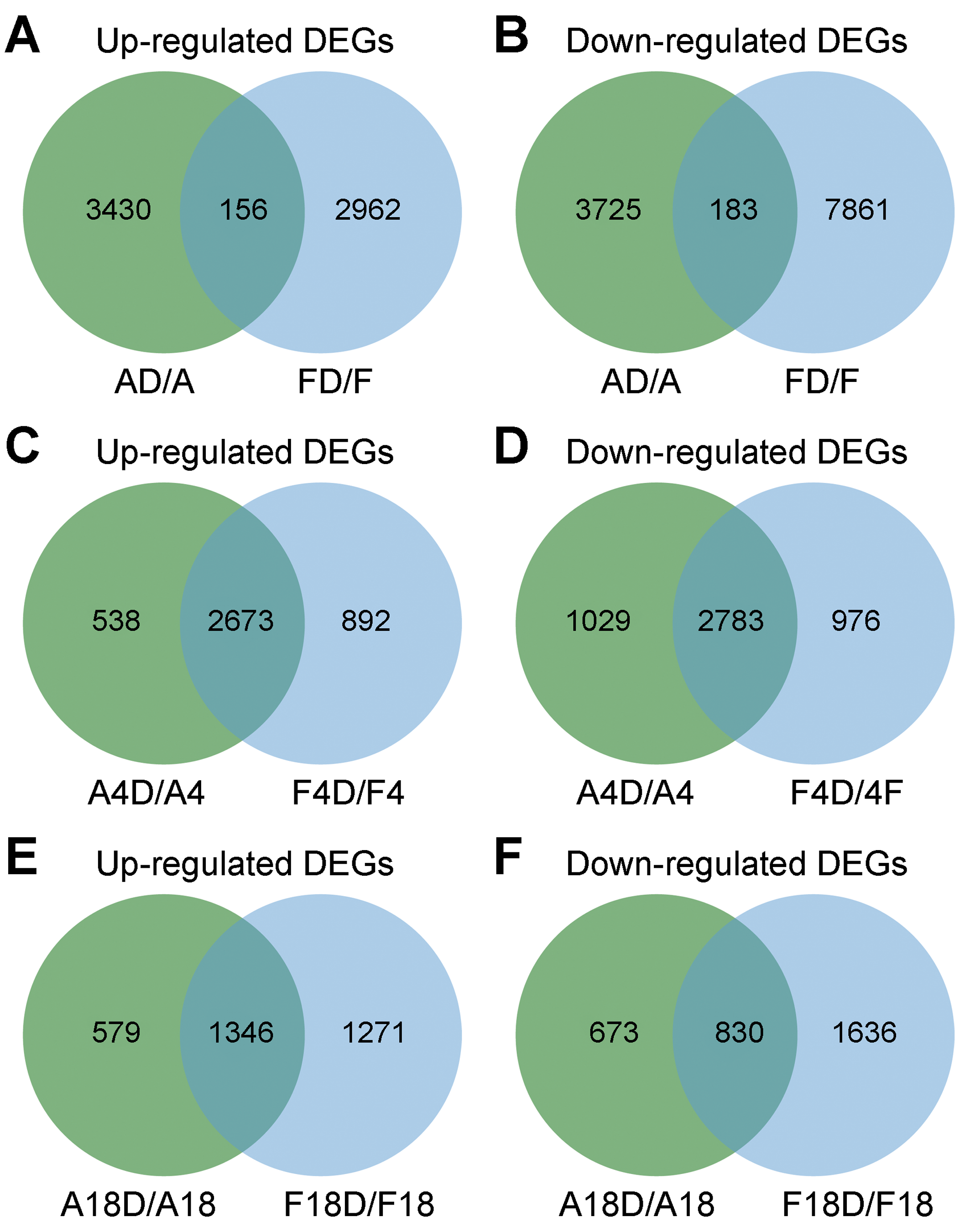

Supplement: S4 Fig — Venn diagrams displaying the overlap of up- (A, C, E) and down-regulated (B, D, F) DEGs between acclimated and non-acclimated fresh plants responding to rapid dehydration stress at each of the three growth stages. (TIF) [file pgen.1009549.s004.tif]

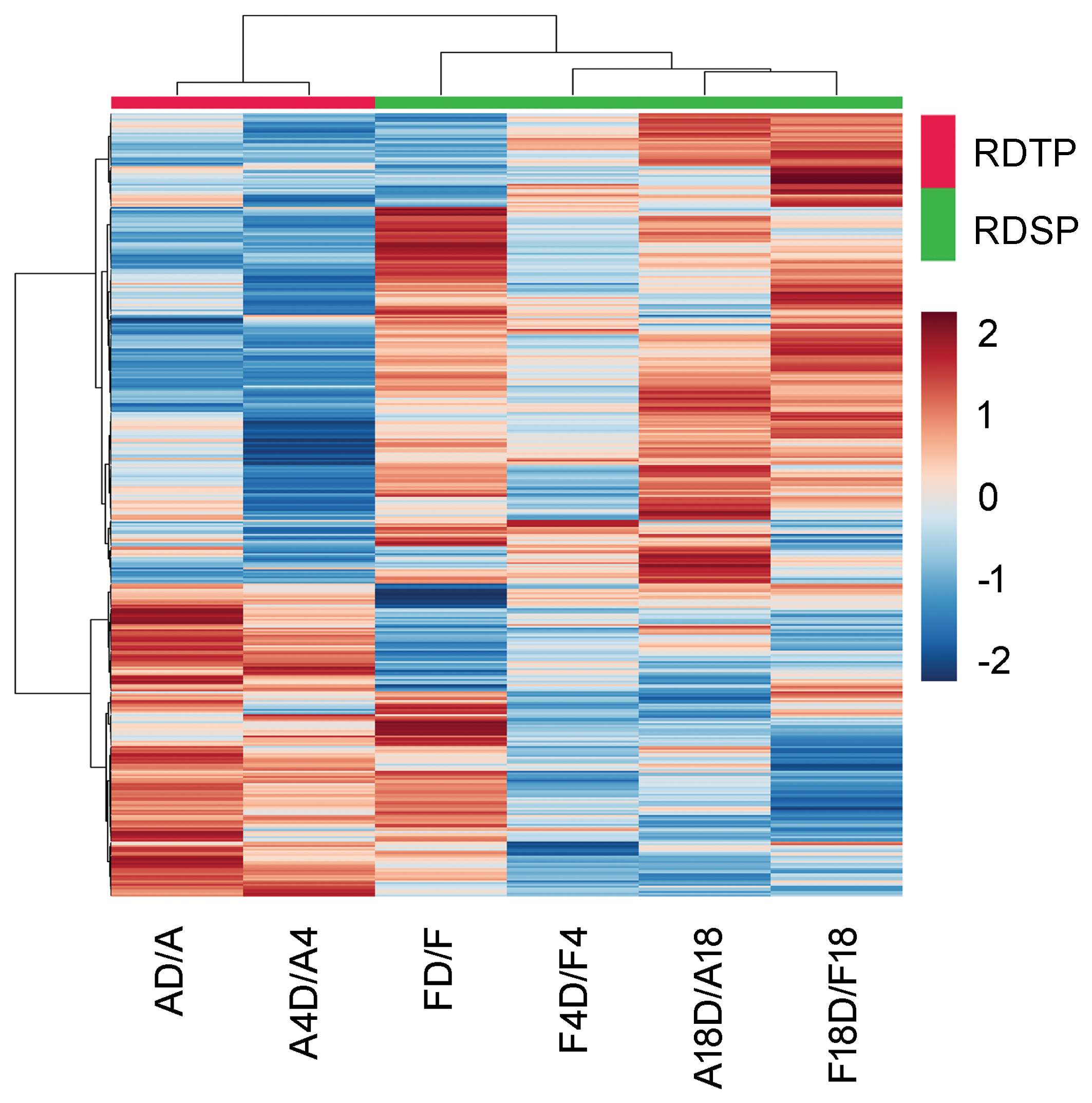

Supplement: S5 Fig — RDTP, rapid dehydration-tolerant plants; RDSP, rapid dehydration-sensitive plants. Scale bar represents log2 fold-change expression (red, upregulation; blue, downregulation) between samples. (TIF) [file pgen.1009549.s005.tif]

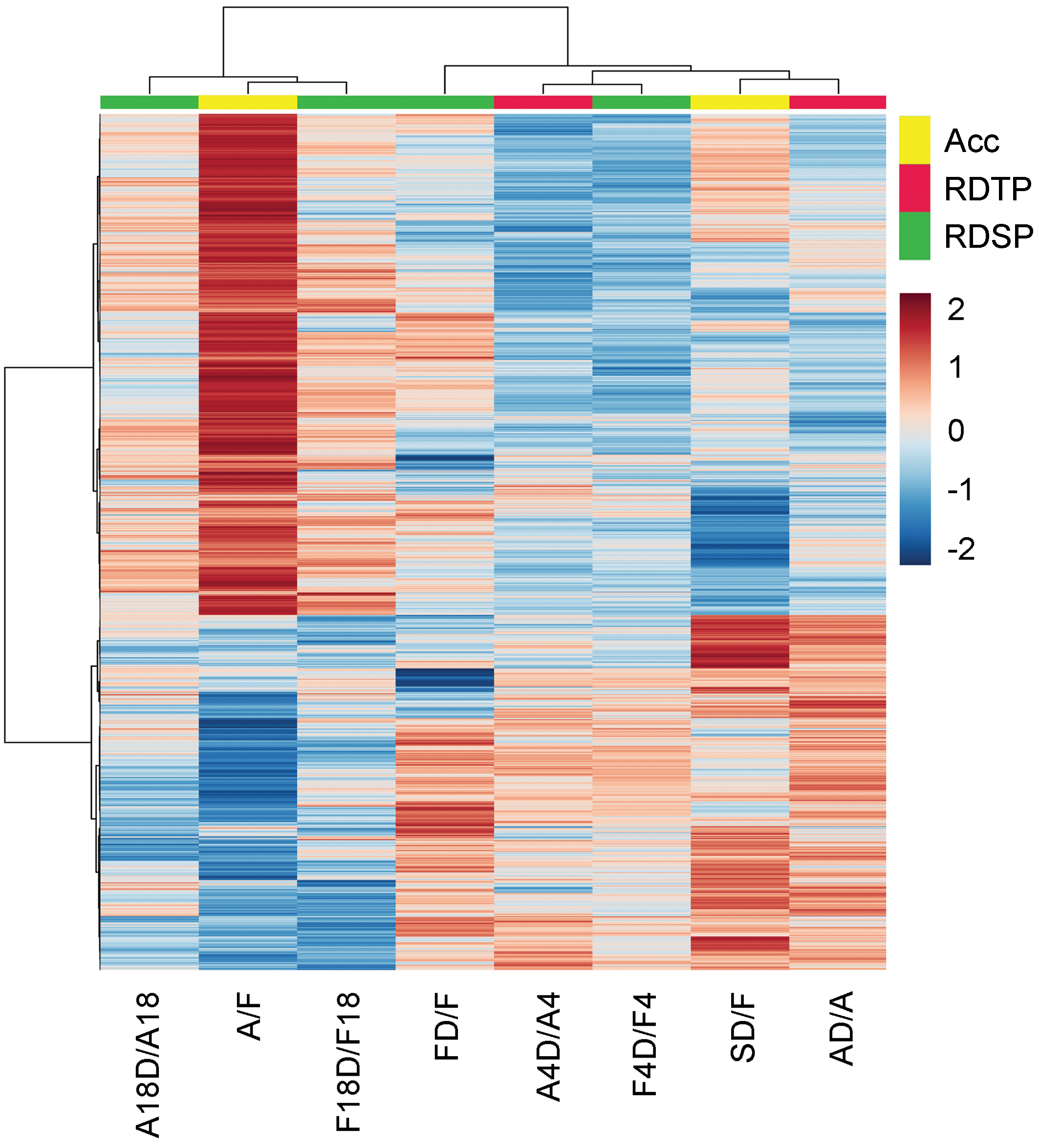

Supplement: S6 Fig — Acc, the acclimation process; RDTP, rapid dehydration-tolerant plants; RDSP, rapid dehydration-sensitive plants. Scale bar represents log2 fold-change expression (red, upregulation; blue, downregulation) between samples. (TIF) [file pgen.1009549.s006.tif]

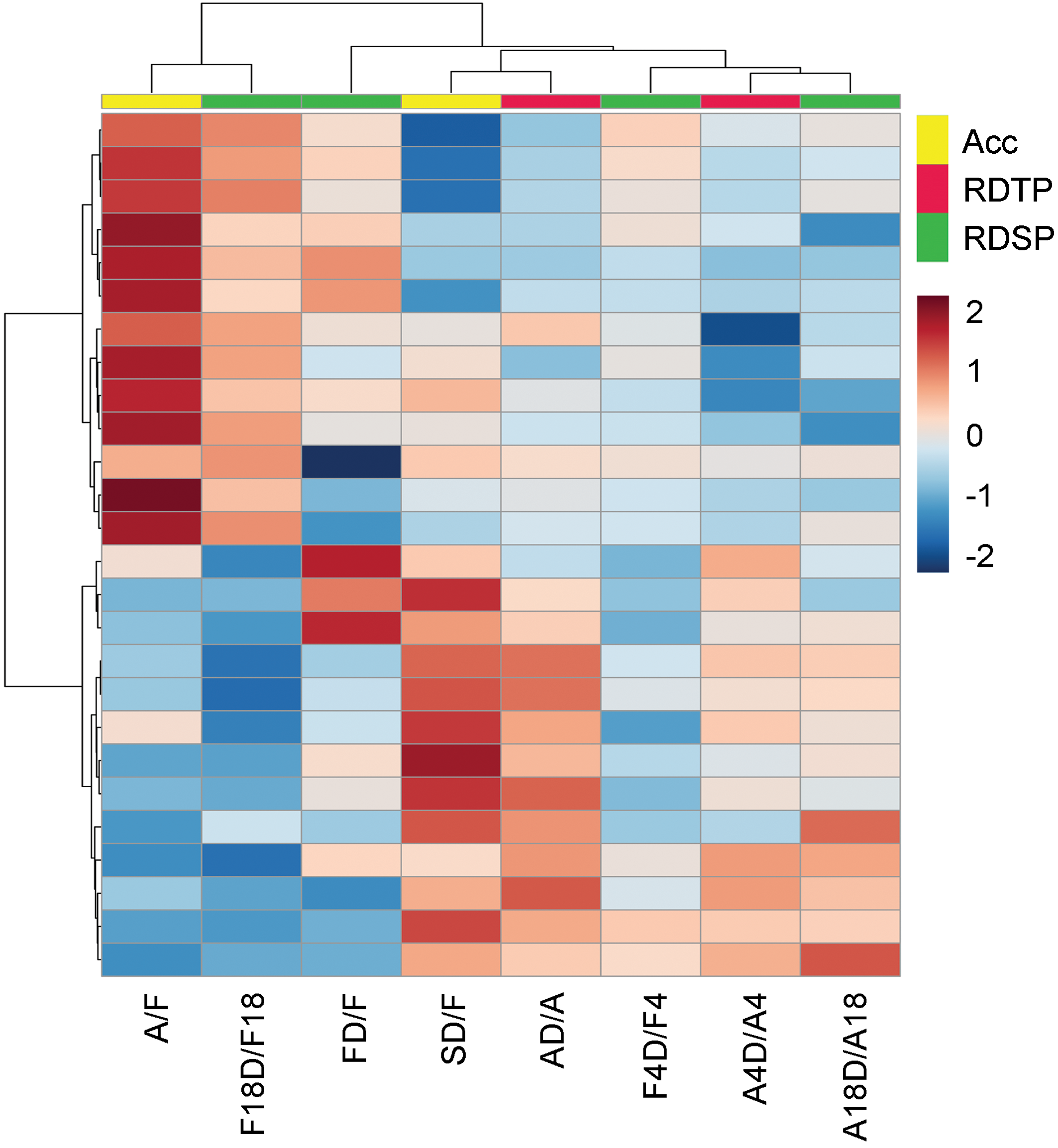

Supplement: S7 Fig — Acc, the acclimation process; RDTP, rapid dehydration-tolerant plants; RDSP, rapid dehydration-sensitive plants. Scale bar represents log2 fold-change expression (red, upregulation; blue, downregulation) between samples. (TIF) [file pgen.1009549.s007.tif]

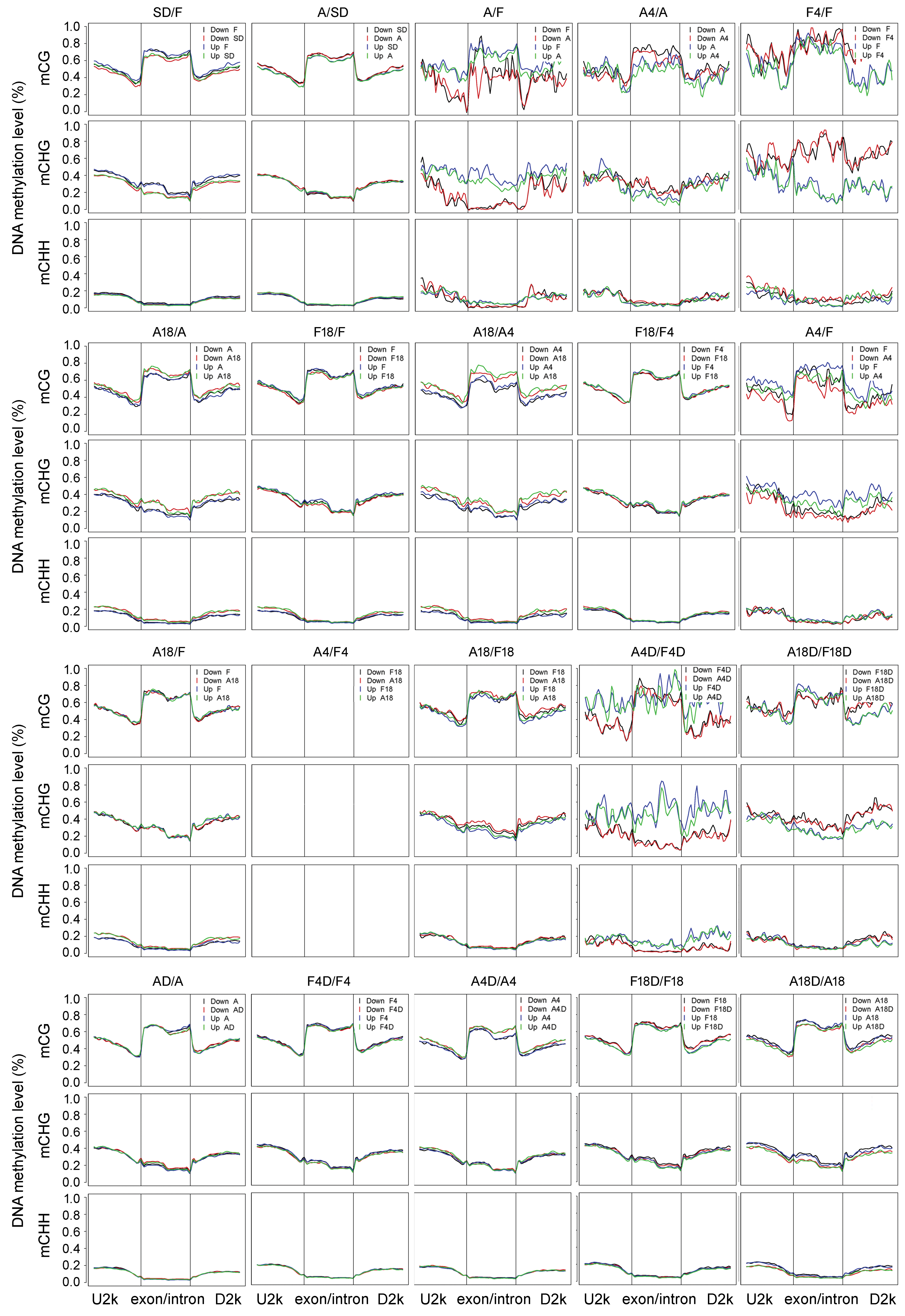

Supplement: S8 Fig — Green and blue lines represent the methylation levels of up-regulated DEGs in the former and latter samples of each comparison, respectively. Red and black lines represent the methylation levels of down-regulated DEGs in the former and latter samples of each comparison, respectively. U2k, 2-kb sequences upstream of the protein-coding region; D2k, 2-kb sequences downstream of the protein-coding region. (TIF) [file pgen.1009549.s008.tif]

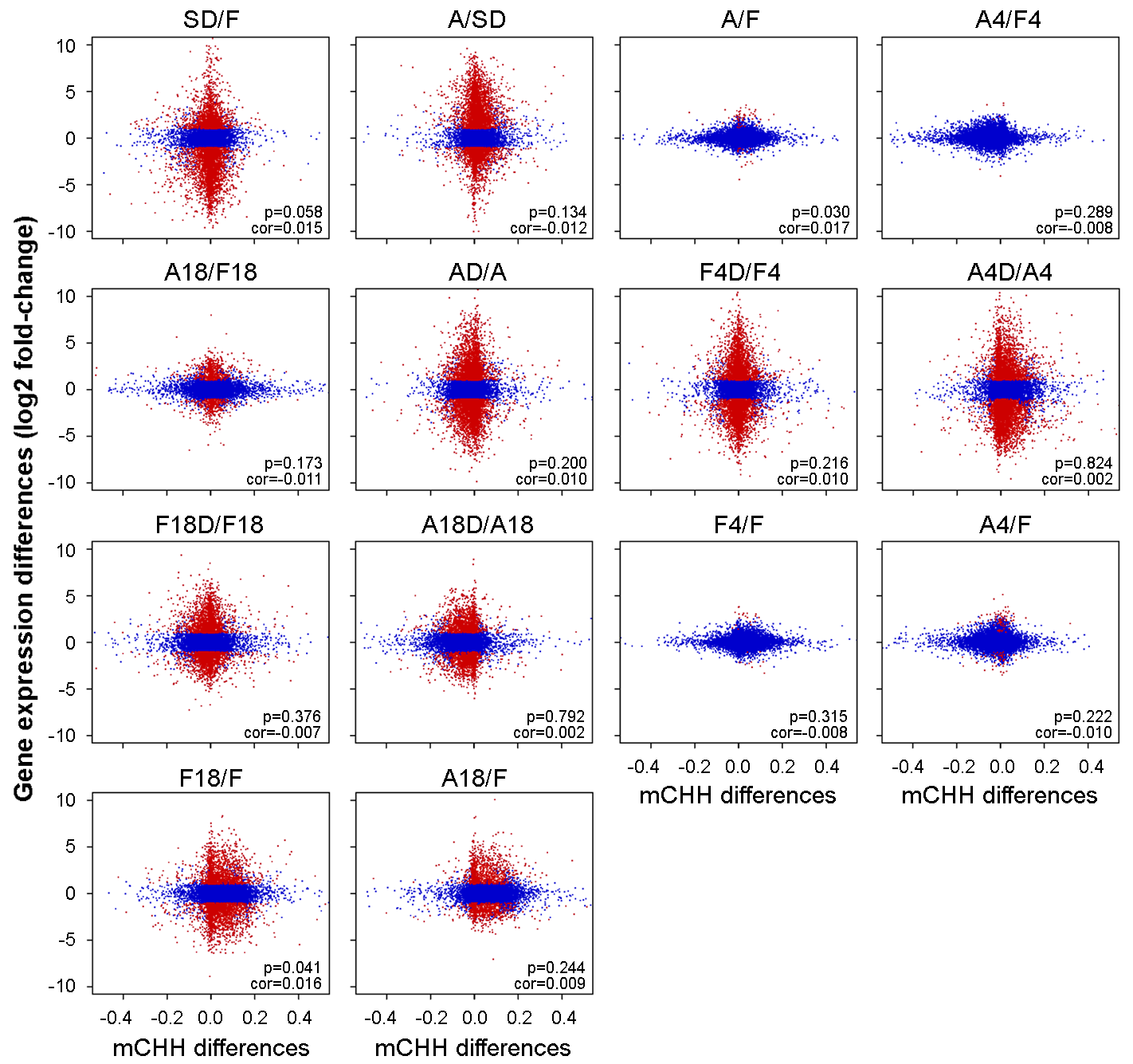

Supplement: S9 Fig — DEGs and non-DEGs are indicated by red and blue dots, respectively. Cor, Pearson correlation coefficient. (TIF) [file pgen.1009549.s009.tif]

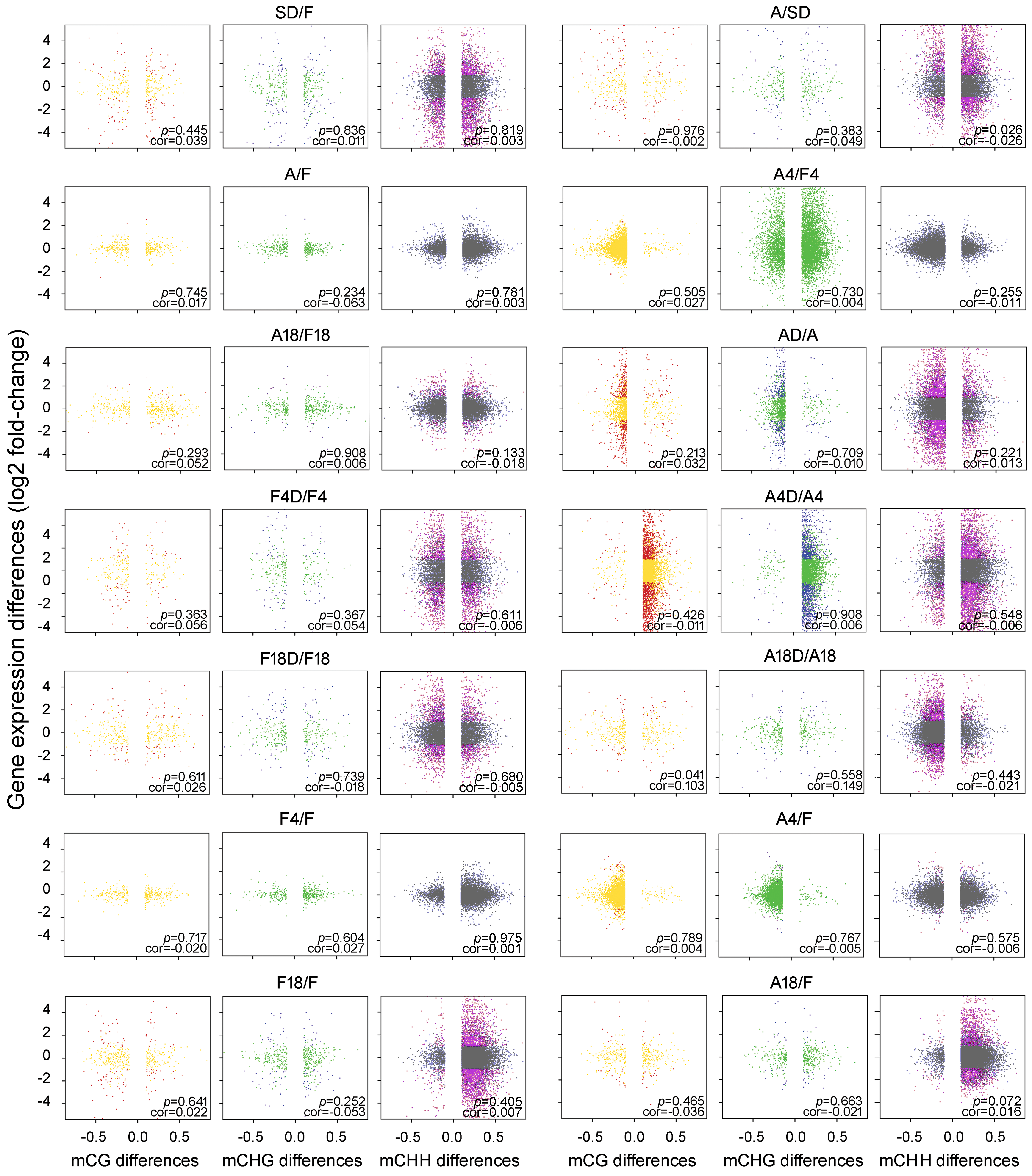

Supplement: S10 Fig — DEGs with CG-, CHG-, and CHH-type of DMRs in their promoter regions are indicated by red, blue, and magenta dots, respectively. Non-DEGs with CG-, CHG-, and CHH-type of DMRs in their promoter regions are indicated by yellow, green, and grey dots, respectively. Cor, Pearson correlation coefficient. (TIF) [file pgen.1009549.s010.tif]

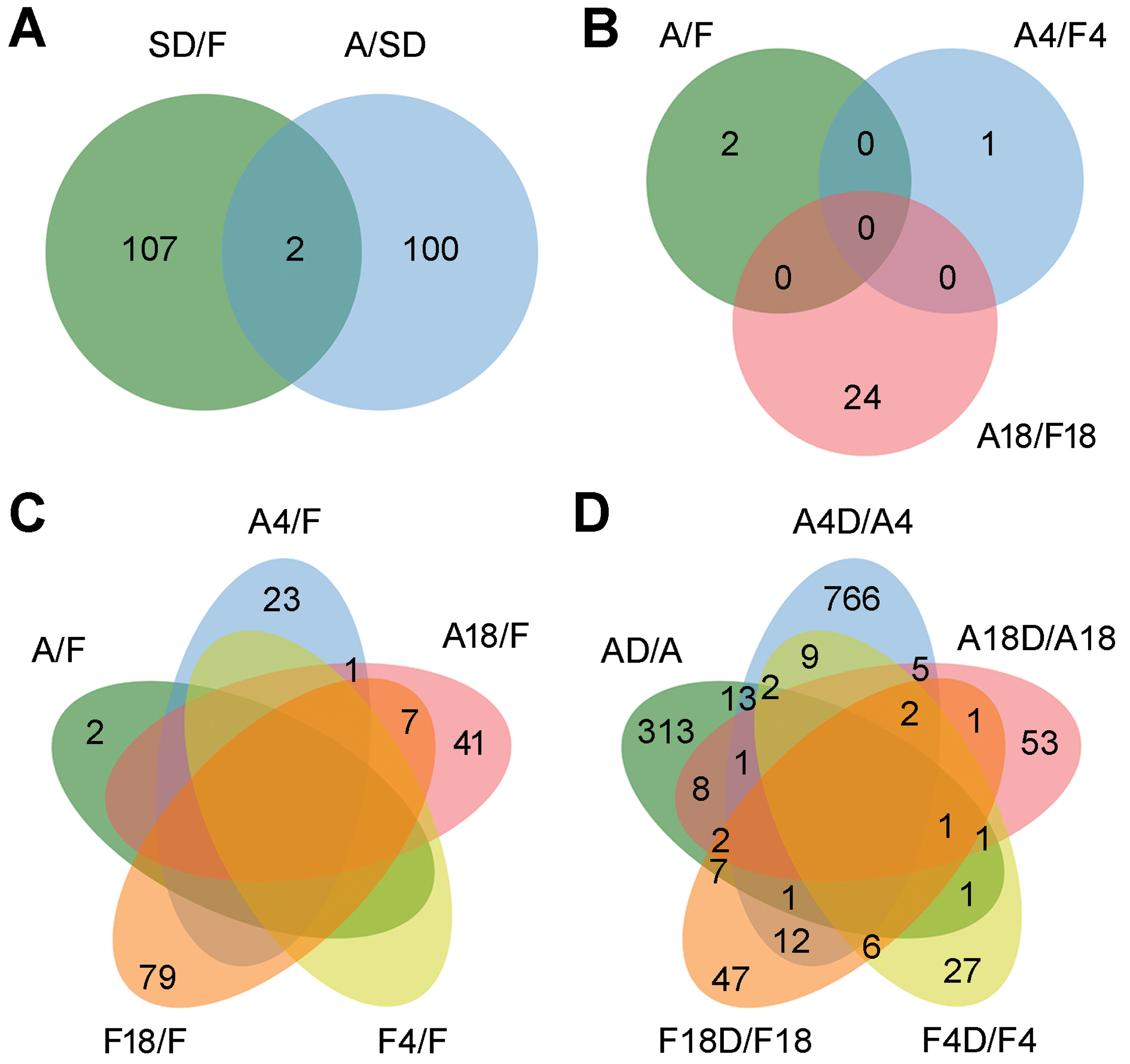

Supplement: S11 Fig — (A) Putative MRGs in plants subjected to slow dehydration stress (SD/F) and rehydration (A/SD). (B) Putative MRGs between acclimated (A, A4, and A18) and non-acclimated (F, F4, and F18) fresh plants. (C) Putative MRGs during plant growth. (D) Putative MRGs in both acclimated and non-acclimated fresh plants subjected to rapid dehydration stress. (TIF) [file pgen.1009549.s011.tif]

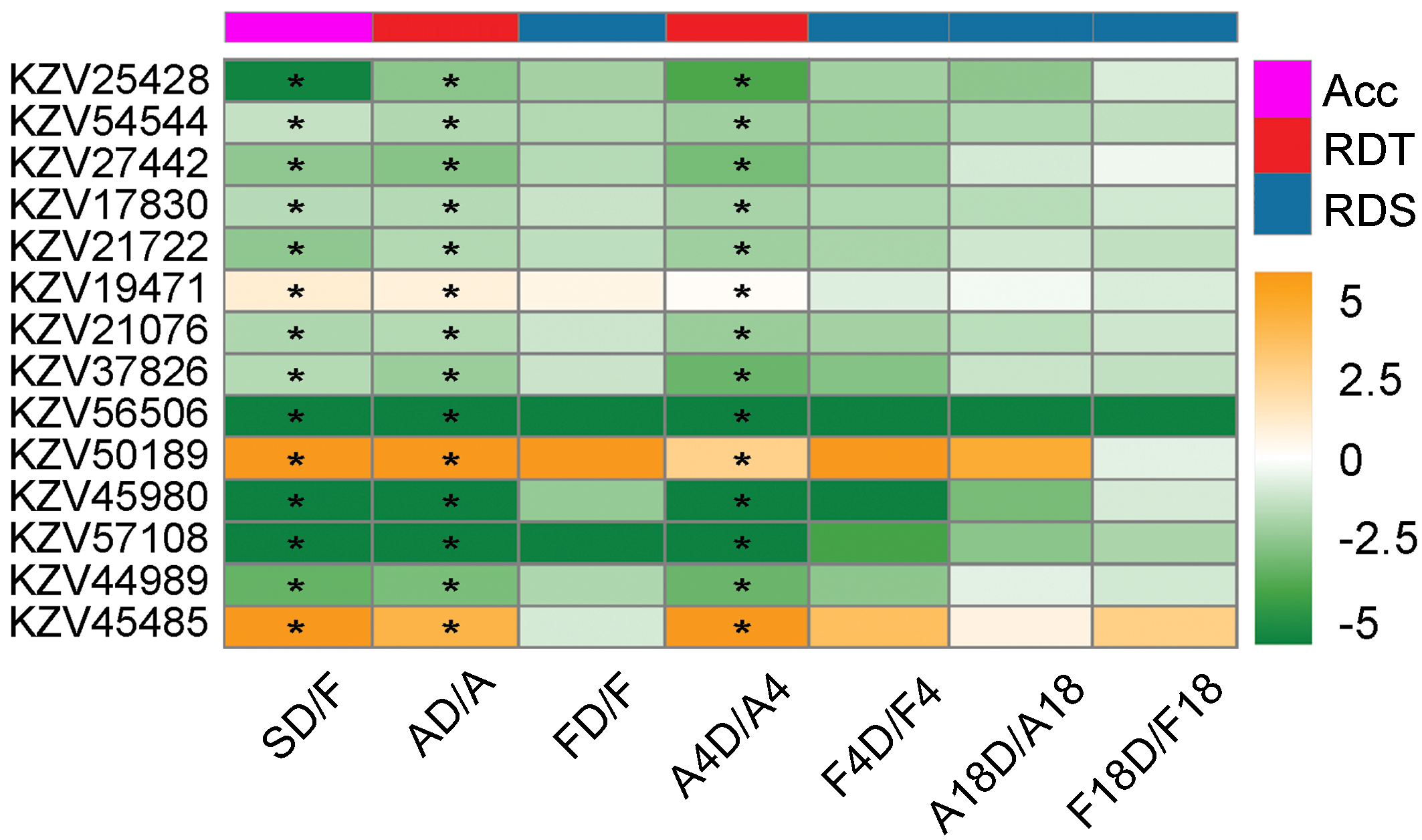

Supplement: S12 Fig — Heatmap showing the expression changes of putative methylation-regulated mid-term dehydration stress memory genes in fresh plants subjected to dehydration stress. Acc, plants subjected to drought acclimation; RDT and RDS represent rapid desiccation-tolerant and -sensitive plants subjected to dehydration stress, respectively. Scale bar represents log2 fold-change expression (yellow, upregulation; green, downregulation) between samples. Genes with significant expression differences (fold change ≥ 2 and adjust p ≤ 0.05) are indicated by asterisks (*) in the squares. (TIF) [file pgen.1009549.s012.tif]
