# Supplementary material for: DNA methylation-mediated modulation of rapid desiccation tolerance acquisition and dehydration stress memory in the resurrection plant Boea hygrometrica
Source: PLoS Genet. 2021 Apr 30;17(4):e1009549. doi: 10.1371/journal.pgen.1009549 (PMC8115786; doi:10.1371/journal.pgen.1009549)
Supplement: Attachment — Submitted filename: Response to Reviewers.docx [file pgen.1009549.s025.docx]

**Response to Reviewers**

# Response to Reviewer #1’s Comments

**Reviewer #1:** The manuscript by Sun et al., is primarily aimed to understand the molecular mechanisms underlying dehydration tolerance, more specifically the mechanism by which drought stress memory is attained in the “resurrection plant” *Boea hygrometrica*. The study starts with an interesting physiologic experiment that proves that the “memory” of drought stress exists in *Boea hygrometrica*, lasting up to 18 weeks.

Then the authors continue the work with the transcriptomic analysis and the profiling of DNA methylation at several steps during acclimation, recovery… but it seems like in their effort to be exhaustive, they describe every figure precisely with no apparent direction nor focus in what they considered important or more specific. This study provides a formidable collection of raw data that has the potential of yielding an exciting output, but one finish the paper with the feeling of these data being under-investigated.

**Reply:** Thank you very much for the positive comments and the valuable constructive criticisms. In the past few weeks, we have substantially revised the manuscript, especially on the analysis and discussion of our transcriptome and methylome data.

In this study, we first demonstrated the drought acclimation-induced dehydration stress memory in the resurrection plant *Boea hygrometrica* based on physiologic measurements (lines 149-173). To elucidate the molecular basis for the acclimation-induced dehydration stress memory in *B. hygrometrica*, we characterized the transcriptome dynamics in *B. hygrometrica* during RDT acquisition, maintenance, and erasing (lines 174-204); three subsets of acclimation pre-induced transcriptional memory genes were identified, suggesting that drought acclimation may contribute to a more rapid and robust response of gene expression changes under rapid dehydration stress (lines 205-266); changes in the expression of DNA methylation pathway genes were observed, indicating the potential involvement of DNA methylation in dehydration stress memory (lines 267-301). Thus, we detected the single base-resolution DNA methylomes (lines 302-359) and conducted correlation analyses between gene expression and DNA methylation within each sample and between gene expression differences and DNA methylation variations between samples (lines 360-448). The results showed that the promoter methylation variations were significantly associated with the differential expression of genes required for fundamental genetic processes during acclimation, growth, and rapid dehydration stress response (lines 402-448). It was also associated with the dehydration stress-induced upregulation of memory genes, including *pre-mRNA-splicing factor 38A*, *vacuolar amino acid transporter 1-like*, and *UDP-sugar pyrophosphorylase*, which may contribute directly or indirectly to the improvement of dehydration tolerance in *B. hygrometrica* plants (lines 449-491).

As you will see in our revised manuscript, additional analyses and explanations of the raw data have been added in the ‘Results’ section, such as “phenotypic responses of the control groups subjected to dehydration stress (lines 156-159)”, “drastic alternations in the transcriptional activity of genes occur during long-term growth and the dehydration stress response (lines 201-204)”, “drought acclimation may contribute to a more rapid and robust response of gene expression changes under rapid dehydration stress (lines 256-258)”, “validation of the RNA-seq data by qPCR (lines 259-266)”, “identification of DNA methylation pathway genes (lines 274-281)”, “acclimation-dependent induction of rapid dehydration stress-responsive genes may be associated with acclimation-related and dehydration-induced changes in DNA methylation (lines 304-310)”, “relationship between the changes in average mC levels and the number of hyper- and hypo-DMRs between samples (lines 345-346)”, “changes of the CG, CHG, and CHH methylation in gene bodies and flanking regions (lines 367-371)”, “functions of CG and non-CG methylation in genic and intergenic regions in gene silencing (lines 385-388)”, and “putative methylation-regulated mid-term dehydration stress memory genes (lines 466-475)”. Meanwhile, some redundant data have been removed in the ‘Results’ section, such as “GO enrichment analysis of dehydration stress memory genes (lines 244-256)”.

In the ‘Discussion’ section, we have added additional discussion of results, combining with numerous previous studies (lines 909-1159), such as “identification of transcriptional memory genes (lines 569-571)”, “transcriptional alternations of methylation-related genes during development and drought stress (lines 583-586)”, “DNA methylation levels in CG, CHG, and CHH contexts in different plants (lines 590-606)”, “reduced DNA methylation levels at symmetrical sites in drought-acclimated plants at an early stage of RDT maintenance (lines 619-627)”, “no significant correlation between transcriptional changes and methylation differences (lines 648-651)”, “involvement of MRGs in genetic information processing and plant purine metabolism (lines 657-662, 666-668)”, and “drought stress-induced hypomethylation and upregulation of memory genes (lines 673-692)”.

The amendments are highlighted in yellow in the “Revised Article with Changes Highlighted”. In addition, we have carefully revised the figures (Figures 2, 6, and 7; S9, S10, and S12 Figs) and English language to make our manuscript more clear and easier to understand. We hope that these changes to the manuscript can provide important and more specific information from our data.

# Response to Reviewer #2’s Comments

**Reviewer #2:** The manuscript by Sun et al., studied the mechanism of acclimation-induced stress memory in Boea hygrometrica by global transcriptome analysis and DNA methylation comparison. It’s very interesting for understanding the resistance mechanism of botany under global climate change. Here we can found that a large number of omics analyses and investigations for uncovering this question have been done. The results and conclusion are reliable and solid, but there are some questions need to be addressed.

Major comments:

- Fig. 3B, the mCG and mCHG level in "A4" is markedly lower than those of other samples. This might be due to the available of only a single biological replicate of WGBS, or the sample-specific expression patterns of the methylation enzymes? The authors should discuss this aspect.

**Reply:** Thanks for pointing out this critical problem. The WGBS library of each treatment was constructed from a pool comprising equal quantities of DNA samples from six biological replicates. Approximately 45 leaves detached from at least 15 individual plants were pooled for DNA extraction in each biological replicate. WGBS yielded an average of 51.01 Gb clean bases (Q30 > 83.24%) from each sample, and approximately 84.35% of reads were mapped to the reference genome, corresponding to an effective depth of 28.96-fold coverage. The number of detected cytosines from each sample reached saturation (see attached **Fig. A1** for WGBS depth and saturation of A4 sample), demonstrating that our sequencing data were sufficient for further analysis. Notably, sharply reduced DNA methylation levels at symmetrical sites but not at non-symmetrical sites were observed in drought-acclimated plants at an early stage of RDT maintenance (Figure 3B), although no significant differences in the transcription of methylation pathway genes were detected in the comparisons of A4/A or A4/F4 (Figure 3A). The hypomethylation status could result from self-repair mechanisms of the physiological recovery from severe dehydration stress that requires the transcriptional activation of substantially more genes (Virlouvet et al., 2018), as evidenced by significantly elevated leaf REC in A4 plants compared with other fresh plants (Figure 1D).

**Fig. A1** WGBS depth and saturation of A4 sample

- line 375, what is the relationship between CHH methylation differences and gene expression changes? I think the authors should analyze and discuss about this, since "larger number of DMRs in CHH context than in CG and CHG contexts was detected" (line 561).

**Reply:** It is true that a larger number of DMRs in CHH context than in CG and CHG contexts was detected between acclimated and non-acclimated plants and in plants responding to dehydration stress (Figure 3D), which might result from the redundancy of the asymmetric CHH motif sequence and less stable of the methylation in that site (Yaish et al., 2018). However, the effect of promoter methylation in the CHH context on gene expression is less than those in CG and CHG contexts, based on the comparison of the transcript abundance between promoter-methylated and -unmethylated genes within each sample (Figure 4B). We have performed the Pearson correlation analysis between gene expression changes and CHH-type methylation variations within their promoter regions during acclimation, growth, and dehydration stress (S9 Fig), as well as the transcriptional changes of genes with different CHH-type methylation levels in their promoter regions and the corresponding promoter CHH methylation differences (S10 Fig). As expected, no significant correlation (*p* > 0.05) between CHH-type methylation variations and gene expression differences was detected during acclimation, growth, or dehydration stress, demonstrating the limited role of mCHH in determining differential gene expression between samples. Corresponding results have been added in the revised manuscript (lines 403-412). We hope they are acceptable.

- line 621, more information about the plant sampling are needed, e.g., maternal genotypes, populations, outcrossed or self-pollinated? Are the collected seeds from genetically independent parents or sib families? All of these information must be addressed.

**Reply:** Seeds of *B. hygrometrica* were collected from a naturally growing population of approximately 200 wild plants inhabiting the Western Hills of Beijing, China (40°12 N, 116°10 E). Most of these plants are dependent on pollination by honeybees, with only a small part of the self-pollination. Therefore, the collected seeds are possibly from both genetically independent parents and sib/half-sib families in the population (Sun et al., 2020). All the collected seeds were mixed in one tube for subsequent experiments. Detailed information on plant sampling has been added in the text (lines 698-702).

- A brief explanation about how did the drought acclimation be done and how long did the dehydration/rehydration treatment last should be mentioned in the Materials and Methods, although references were cited.
**Reply:** For drought acclimation treatment, three-month-old seedlings of *B. hygromtrica* were subjected to slow-onset water deprivation treatment by withholding water for 14 d (slow soil-drying) after saturating soil moisture, and then re-watered for 3 d (Zhu et al., 2015; Sun et al., 2018). Untreated plants served as the non-acclimated control group. Rapid dehydration (rapid air-drying) treatment was imposed by transferring plants from soil to Petri dishes and drying immediately at 25°C and 60% RH for 2 d. The air-dried plants were rehydrated by subjecting them to wet filter paper in Petri dishes for 3 d. Both drought-acclimated and non-acclimated plants were subjected to rapid air-drying and rehydration after continuous cultured for 0, 4, 8, 13, 18, and 22 w, respectively (Figure 1A). Detailed information about drought treatment has been added in the text (lines 702-714).

Minor comments:

- Line 23, incomplete presented.

**Reply:** Sorry for our carelessness. The sentence has been revised (lines 22-26), and the mistake has been corrected.

- Line 155, the result of statistical analysis (P-value?) is missing. It would be important clarify "significant" in the text.

**Reply:** We have added the *p*-value of each statistical analysis in the text (lines 162, 167, 213, 397, 410, etc.).

- Line 203, differentiate between "rapid desiccation-tolerance (RDT)" and "rapid dehydration tolerance (RDT)".

**Reply:** To avoid confusion, both “rapid desiccation-tolerance (RDT)” and “rapid dehydration tolerance (RDT)” have been changes to “rapid desiccation tolerance (RDT)” in the title, abstract, main text, and figure legends. In addition, the phrase “RDT plants” has been changed to “rapid desiccation-tolerant plants” in the text.

- Fig. 1, what do the error bars represent in the plots?

**Reply:** Data are presented as the mean ± standard error of four to six biological replicates (each replicate with 6 plants), and different superscript letters over bars represent statistical differences at the 0.01 level. Error bars represent standard errors. Accordingly, the corresponding description has been added in the figure legend.

- Figs. 3A, 6D, what do the asterisks represent in the heatmaps? Not clear.

**Reply:** In Fig. 3A, genes with significant expression changes (fold change ≥ 2 and adjusted *p* ≤ 0.05) in drought-acclimated plants compared with the non-acclimated control groups (A/F, A4/F4, and A18/F18) and in plants subjected to dehydration stress (SD/F, FD/F, A/F, F4D/F4, A4D/A4, F18D/F18, and A18D/A18) are indicated by asterisks (*) in the corresponding squares. In Fig. 6D (changed to Fig. 7D), significant expression changes (at the 0.05 level) of genes in 5-azaC/SAM treated plants compared with the untreated control group are indicated by asterisks (*) in the corresponding squares. Corresponding descriptions have been revised in the figure legends.

- Supplemental figure and table legends are not self-explanatory. More details should be given.

**Reply:** Details of the supplemental figures and tables have been added in their corresponding legends. We hope they are acceptable. Thanks again for the reviewer’s valuable comments and suggestions to our manuscript.

**Main references:**

Sun R-Z, Lin C-T, Zhang X-F, Duan L-X, Qi X-Q, et al. (2018) Acclimation-induced metabolic reprogramming contributes to rapid desiccation tolerance acquisition in *Boea hygrometrica*. Environ Exp Bot. 148: 70-84.

Virlouvet L, Avenson TJ, Du Q, Zhang C, Liu N, et al. (2018) Dehydration stress memory: gene networks linked to physiological responses during repeated stresses of Zea mays. Front Plant Sci. 9: 1058.

Yaish MW, Al-Lawati A, Al-Harrasi I, Patankar HV (2018) Genome-wide DNA Methylation analysis in response to salinity in the model plant caliph medic (Medicago truncatula). BMC Genomics. 19: 78.

Zhu Y, Wang B, Phillips J, Zhang Z-N, Du H, et al. (2015) Global transcriptome analysis reveals acclimation-primed processes involved in the acquisition of desiccation tolerance in *Boea hygrometrica*. Plant Cell Physiol. 56: 1429-1441.

# Response to Reviewer #3’s Comments

**Reviewer #3:** 1. The authors did the qPCR of 5-azaC/SAM treated *B. hygrometrica* seedlings and BS-PCR for addressing the role of DNA methylation with RDT. However, they didn't validate the results from transcriptomes by qPCR or any other method.

**Reply:** Thanks for the reviewer’s comments. To validate the RNA-seq data, the relative expression levels of 10 genes randomly selected from the lists of up- and down-regulated RDT-specific DEGs were determined in each sample by quantitative real-time PCR (qPCR). The gene expression levels are presented as the mean ± SD by the Ct method using *elongation factor 1α* and *18S rRNA* as the reference genes. The gene-specific primers used for qPCR are listed in S11 Table. Three biological replicates were analyzed using the mean values of three technical replicates for each sample. Results showed that the transcription profile of these genes in both drought-acclimated and non-acclimated plants during growth and dehydration stress obtained from an independent qPCR evaluation was significantly positively correlated [Pearson correlation coefficient (cor) = 0.733, *p* < 0.001] with those from the transcriptomes (Figure 2F), indicating the high reproducibility of the RNA-Seq results. Corresponding results have been added in the text (lines 259-266).

2. The language aspect needs to be refined.

**Reply:** We have carefully proofread the manuscript and corrected some grammar errors. Meanwhile, the language has been checked by two professional editors, both native speakers of English. As you will see, major amendments have been made in the “Revised Article with Changes Highlighted”, which are highlighted in yellow. We hope that with these modifications and improvements, our revised manuscript will be more clear and easier to understand.
